# SHRINE: Enabling Nationally Scalable Multi-Site Disease Studies

**DOI:** 10.1371/journal.pone.0055811

**Published:** 2013-03-07

**Authors:** Andrew J. McMurry, Shawn N. Murphy, Douglas MacFadden, Griffin Weber, William W. Simons, John Orechia, Jonathan Bickel, Nich Wattanasin, Clint Gilbert, Philip Trevvett, Susanne Churchill, Isaac S. Kohane

**Affiliations:** 1 Center for Biomedical Informatics, Harvard Medical School, Boston, Massachusetts, United States of America; 2 Children's Hospital Informatics Program, Children's Hospital Boston, Boston, Massachusetts, United States of America,; 3 i2b2 National Center for Biomedical Computing, Brigham and Women's Hospital, Boston, Massachusetts, United States of America; 4 Bioinformatics Program, Boston University, Boston, Massachusetts, United States of America; 5 Partners Healthcare System, Research Computing, Boston, Massachusetts, United States of America; 6 Massachusetts General Hospital, Boston, Massachusetts, United States of America; 7 Beth Israel Deaconess Medical Center and Harvard Medical School Information Technology, Boston, Massachusetts, United States of America; 8 Clinical Research Information Technology, Dana-Farber Cancer Institute, Boston, Massachusetts, United States of America; 9 Information Systems Department, Children's Hospital Boston, Boston, Massachusetts, United States of America; University of Western Australia, Australia

## Abstract

Results of medical research studies are often contradictory or cannot be reproduced. One reason is that there may not be enough patient subjects available for observation for a long enough time period. Another reason is that patient populations may vary considerably with respect to geographic and demographic boundaries thus limiting how broadly the results apply. Even when similar patient populations are pooled together from multiple locations, differences in medical treatment and record systems can limit which outcome measures can be commonly analyzed. In total, these differences in medical research settings can lead to differing conclusions or can even prevent some studies from starting. We thus sought to create a patient research system that could aggregate as many patient observations as possible from a large number of hospitals in a uniform way. We call this system the ‘Shared Health Research Information Network’, with the following properties: (1) reuse electronic health data from everyday clinical care for research purposes, (2) respect patient privacy and hospital autonomy, (3) aggregate patient populations across many hospitals to achieve statistically significant sample sizes that can be validated independently of a single research setting, (4) harmonize the observation facts recorded at each institution such that queries can be made across many hospitals in parallel, (5) scale to regional and national collaborations. The purpose of this report is to provide open source software for multi-site clinical studies and to report on early uses of this application. At this time SHRINE implementations have been used for multi-site studies of autism co-morbidity, juvenile idiopathic arthritis, peripartum cardiomyopathy, colorectal cancer, diabetes, and others. The wide range of study objectives and growing adoption suggest that SHRINE may be applicable beyond the research uses and participating hospitals named in this report.

## Introduction

Results of medical research studies are often contradictory[1], [2] or cannot be reproduced[3], [4], [5], [6], [7]. One reason is that there may not be enough available patient subjects[8] observed over a long enough time period[9], [10]. Another reason is that patient populations may vary considerably across geographic[11] and demographic boundaries[12] thus limiting how broadly the results apply. Even when similar patient populations are pooled together from multiple locations, differences in medical treatment[13] and record systems[14], [15] can limit which outcome measures can be commonly analyzed. In total, these differences in medical research settings can lead to differing conclusions or can even prevent some studies from starting.

Consider Acute Lymphoblastic Leukemia (ALL), a rare pediatric cancer. Since each hospital only sees a few cases per year, studies of clinical effectiveness or disease biology are only realistically possible through multi-center analyses[16]. Now consider type 2 diabetes, a common polygenic disease having many risk factors[17] and comorbid diagnoses[18], [19]. The number of adults in the United States with newly diagnosed diabetes has more than tripled since 1980 [20] affecting patient populations at different rates[21] among states [11], ethnicities [12], and socioeconomic positions[12]. Grouping populations of diabetic patients according to demographics, disease risk, and previous treatments results in many smaller sets of patients to analyze. Thus, even for a disease reaching epidemic levels it is often necessary to observe multiple health care systems in parallel to study enough patients representing the general population.

We thus sought to create a patient research system that could aggregate as many patient observations as possible from as many hospitals as possible. We call this system the ‘Shared Health Research Information Network’, with the following properties: (1) reuse electronic health data from everyday clinical care for research purposes; (2) respect patient privacy and hospital autonomy; (3) aggregate patient populations across many hospitals to achieve statistically significant sample sizes that can be validated independently of a single research setting; (4) harmonize the observation facts recorded at each institution such that queries can be made across many hospitals in parallel; (5) scale to regional and national collaborations.

The purpose of this report is to provide open source software[22] for multi-site clinical studies and to report on early uses of this application. At this time SHRINE implementations have been used for multi-site studies of autism co-morbidity[23], juvenile idiopathic arthritis[24], peripartum cardiomyopathy[25], colorectal cancer, diabetes[26], and likely others. The wide range of study objectives and growing adoption of the software suggest that SHRINE may be applicable beyond the research uses and participating hospitals named in this report.

## Results

SHRINE has been developed and deployed to at least six networks in the United States serving a wide range of study interests (Table 1). On the east coast, 5 Harvard affiliated teaching hospitals are now able to query and analyze anonymized data on over 6 million patients covering a 10 year period. Authorized investigators perform Boolean searches for patient populations matching detailed study criteria including patient demographics, diagnoses, medications, and common lab tests. The east coast network at Harvard has been used to conduct the largest study to date of co-morbidities in Autism Spectrum Disorders[23]. The Harvard network was also used to help validate a novel discovery in peripartum cardiomyopathy[25], Many other population scale studies are now possible for 7500+ authorized Harvard users. On the west coast, 3 independent academic medical centers have utilized SHRINE for an evaluation study focused on Type II Diabetes[26]. Nationally, SHRINE has been used to link 61 health institutions to create the largest US patient registry of pediatric rheumatic diseases[24], [27]. Another national SHRINE project is in development spanning 9 large US institutions for studies of autism and diabetes. In Europe, a consortium spanning 5 countries is evaluating the use of SHRINE for use in clinical trials and medication safety[28], [29]. The research objectives, policy agreements, and technical systems of each SHRINE network exhibit a high degree of heterogeneity, suggesting that this approach is broadly applicable for a wide range of patient studies.

**Table 1:** Deployed SHRINE networks.

Location	# Institutions	#Patients	Research Focus
SHRINE East	5	6.1 M	Any
National Disease Registry	61	∼5,000	Pediatric Rheumatic Diseases
National Demo	9	1.6 M+	Autism, Diabetes
California State	5	∼11 M	Diagnoses, Procedures
SHRINE West	3	4.2 M+	Diabetes Epidemiology

The Harvard implementation (SHRINE east) is non-disease specific network used by faculty and fellows. Some studies have been completed and published. The National disease registry is the largest disease registry in the US of its kind. The National Demonstration network is being used to analyzing co-morbidities of autism spectrum disorders and diabetes in geographically disperse US states. Lastly, HMO SHRINE was a HMORN pilot project with 12 M+ patients. HMO SHRINE is not listed here because the pilot was completed successfully.

### Availability

SHRINE is freely available Open Source Software [22].

## Methods

### I. Design and Implementation

The goal of SHRINE is to query large, independent patient populations to address problems of insufficient sample size and sample bias. SHRINE is designed to reuse information captured during patient care[30], [31], to protect patient privacy[32], to query heterogeneous health systems simultaneously, and to scale to nation-wide participation[33]. SHRINE aims to serve multiple study needs such as cohort discovery[34] and population scale measurements[35], [36].

The proof of concept system at Harvard was implemented during Summer 2008 with a single year of patient demographic and diagnosis data with access limited to users responsible for building and demonstrating the system[37]. The production peer-to-peer (P2P) system has since been developed and provides federated user identity, asynchronous query broadcast and aggregation, scalable network topologies, and tools for mapping between medical concept coding systems.

### II. Investigator Scenario

**Figure 1:**
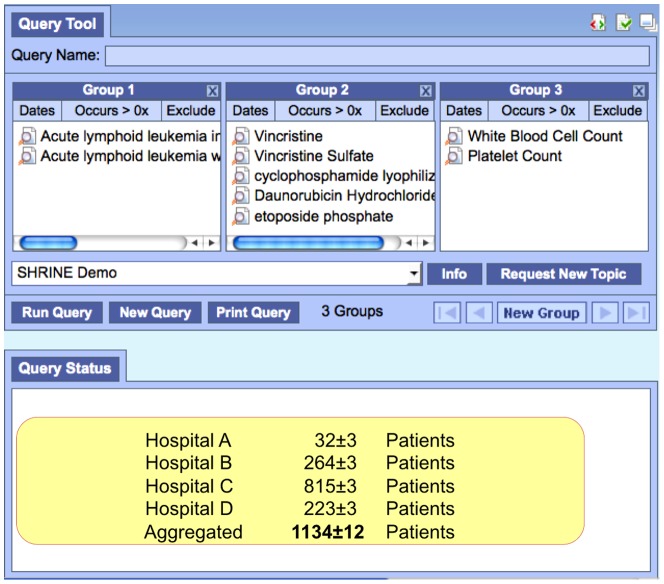
Investigator's perspective of the SHRINE Webclient. *Group 1* defines searches for patients with Acute Lymphoid Leukemia (ALL). *Group 2* refines the search result to only those patients having one of the medications listed. The medications shown are all chemotherapeutic agents administered during intensive phase. *Group 3* further refines the result to require a lab test administered during diagnosis. Lab test values can be set directly or flagged as ‘abnormally high/low’. In the Query Status window, patient counts are displayed with a Gaussian blur to provide additional privacy safeguards of small patient populations. Results are shown for each hospital and the aggregated patient set size.

An Investigator at Children's Hospital Boston is interested in finding patients with Acute Lymphoid Leukemia (ALL) to study the effectiveness of different chemotherapeutic agents in children and adults (Figure 1). Because the incidence of ALL is rare, she needs to aggregate patients from many hospitals to achieve statistical significance. She applies for access to SHRINE, which certifies that she is a qualified faculty member of a participating hospital and has received query approval from the local Data Steward. Her query for ‘Acute Lymphoid Leukemia’ (with or without mention of remission) is then broadcasted to each one of the participating hospitals and she is returned the aggregated patient sets. She further refines her query to only include patients treated with a multidrug chemotherapy regimen, as well as a complete blood count test to confirm the ALL diagnoses. She then requests IRB approval for access to the identified patient cohort. Using SHRINE, she finds potentially five times as many patients than if she looked only at a single hospital. Importantly, the aggregated cohort contains both pediatric and adult cases necessary to conduct this leukemia study.

### III. Federated Query Sequence

From the investigator user perspective, SHRINE queries multiple hospitals at the same time and aggregates results that match the study criteria (Figure 1). From the system perspective, SHRINE is a peer-to-peer (P2P) network of independently controlled ‘peer’ databases. In SHRINE, there is no centralized authority or centralized database – each hospital verifies their own investigator employees, protects their own patient subjects, and hosts their own database of observation facts.

**Figure 2:**
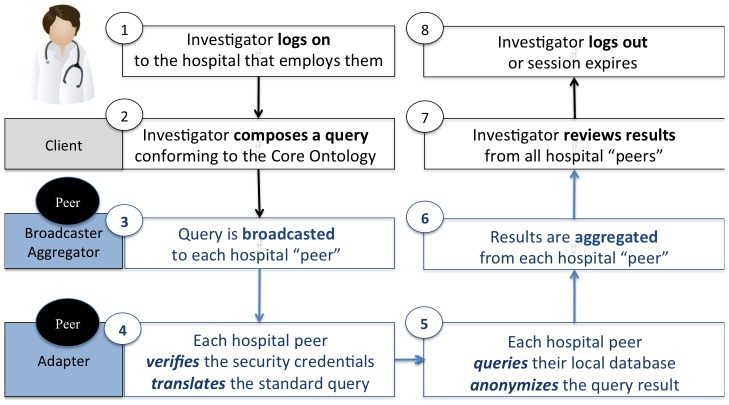
Federate Query Sequence. The investigator logs in and composes a query in steps 1–2. SHRINE securely queries multiple hospital peers and returns aggregated results in steps 3–6. The process of securing and translating queries across multiple hospitals is invisible to the investigator user. Lastly, the investigator reviews the results and logs out in steps 7–8.

First, the investigator must login to the hospital that employs them. All investigator queries are digitally ‘signed-by’ their employer in accordance with policy agreements. Second, the investigator composes a query that conforms to the SHRINE Core Ontology. The Core Ontology defines the standard set of medical concepts and hierarchical relationships that can be used to compose a query. Third, the query is broadcasted to each ‘peer’ hospital. Every hospital peer must have prior regulatory approvals and business agreements. Fourth, each peer verifies that the incoming query is from a trusted broadcaster and translates the incoming query to be executed on the local patient data repository. Fifth, each peer queries their local patient data repository and anonymizes the query result. Finally, results are aggregated and presented to the investigator (Figures 2 and Figure S1).

The following sections describe how to compose a patient query using standard medical ontologies, how to secure patient privacy, how to prepare data mappings, how to translate federated queries, and lastly, how to scale the network to nationwide participation.

### IV. Composing a Patient Query

Patient queries are composed using concepts and relationships defined in an ontology. The SHRINE *Core Ontology* supports many concepts recorded during patient care including diagnoses, medications, lab tests, and demographics (Table 2). The *Core Ontology* contains 13,000+ diagnosis concepts and 4,500+ drug ingredient concepts[38].

**Table 2:** SHRINE Core Ontology.

CATEGORY	CODING SYSTEM	HIERARCHY
***Diagnoses***	ICD-9-CM	CCS2
***Medications***	RxNorm	NDF-RT
***Lab Tests***	LOINC	
***Demographics***		
* Gender*	HL7 Administrative Gender	
* Language*	ISO 639-1	
* Marital Status*	HL7 Marital Status	
* Race and Ethnicity*	CDC Race & Ethnicity Code Sets	
* Religion*	HL7 Religious Affiliation	

*Left column*: categories supported in the core ontology include diagnoses, medications, lab tests, and demographics. *Middle column*: coding system used for each category. The demographics category uses multiple coding systems to handle the relevant sub-categories such as gender and language. *Right column*: hierarchy used to group medically related concepts. Standard hierarchies were adopted where possible, which was the case for diagnoses and medications.

**Figure 3:**
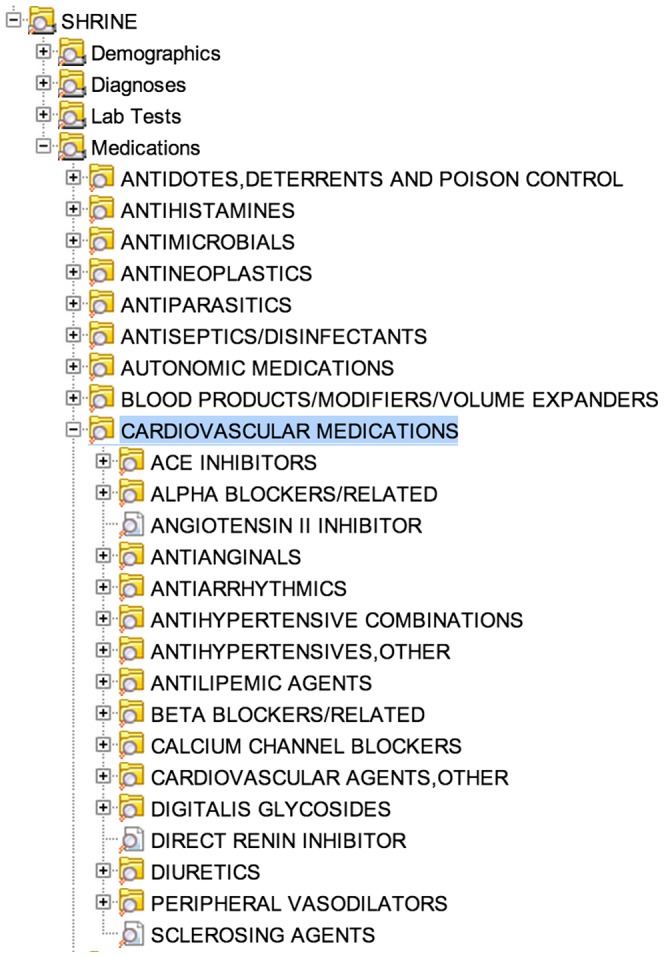
Query Expansion in the Core Ontology. *Selected Example*: ‘Cardiovascular medications’ is selected and the child contents are shown. At runtime, the query is expanded to include every concept in the cardiovascular medication group, recursively.

Hierarchical relationships[38], [39] are used to organize the vast number of medical concepts into groups that are easier for an investigator to query and analyze. Consider heart disease, the leading cause of death in the US[40]. Heart disease has many billable conditions recorded during care delivery – 40 codes just for various episodes and subtypes of heart attack (Acute Myocardial Infarction). Patients with heart disease may also use a beta-blocker, ACE inhibitor, or other cardiovascular medication. Using the hierarchy makes it easier to query medically related medications and diagnoses (Figure 3). SHRINE currently supports a subset of the patient query features available in i2b2: Boolean concept operators (and, or, not), hierarchical paths (query expansion), and observation constraints (dates, number of occurrences).

Composing a patient query is usually an iterative process that begins with a single large patient set and proceeds by analyzing several smaller patient sets. First, an investigator may wish to see if there are enough cases and controls to power their study. Second, the investigator may refine the query criteria to additionally require study features such as co-morbid diagnoses, medication prescriptions, and lab tests. Third, the investigator may subdivide patient sets according to age group, gender, or other demographic criteria. Each investigator is free to compose queries that match their study objectives, receiving query answers in seconds that would otherwise take days or even years to obtain.

### V. Securing Patient Privacy

#### Hospitals are stewards of patient privacy

Striking the balance between research benefit and disclosure risk is a challenging responsibility for each hospital Institutional Review Board (IRB). Given that each hospital is responsible for protecting the privacy of their patients, it follows that each hospital should retain the authority to approve or reject requests for access to data on their patients. When the request comes from an investigator employed by the hospital, it is reasonable to assume the hospital knows who the investigator is and can verify her identity. However, when the request is from an investigator at a different hospital, how can the investigator be credentialed and trusted?

#### Technical solutions for building trust between hospital peers

Trust agreements between collaborating SHRINE peer institutions are formalized through mutual exchange of X509 digital certificates. SHRINE uses digital certificates to secure HTTPS communication and to identify hospital peers[41]. When an investigator ‘logs-on’ at their hospital, the employing hospital certifies the employee credentials and digitally signs the identity of the investigator. The digital signature[42] is attached to the query criteria before the query is sent (broadcast) to every trusted peer in the network. When the query is received the source is verified before processing. If the signature is from an untrusted source or if the signature is invalid due to identity tampering, then the query is rejected. Because digital signature verification is a local operation, hospital credentialing systems do not need to be exposed to other institutions.

To further protect against external hacking attempts, institutional firewalls at each hospital are configured to allow only IP addresses of trusted peer institutions. To further protect against internal privacy accidents, population statistics should be used until the time that individual patient facts are truly necessary for the study. The default level of data access in SHRINE is ‘anonymized’ meaning that only the size of the patient set is returned, not a line item list of patient details. SHRINE anonymized results are further obfuscated to protect very small populations (<10 patients). Accidental sharing of patient numbers poses little or no risk to patient privacy. If additional permission has been granted by the hospital IRB, additional data access may be provided by the hospital to authorized investigators.

#### Joining the Network

Prior to joining a SHRINE network, each hospital secures institutional and regulatory approval. This includes an IRB review (which may be expedited if the SHRINE queries are only for aggregate numbers of patients meeting criteria). It also requires agreement on a set of operational principles or ‘Business Rules’ by the leadership of participating institutions. The Business Rules (*those implemented at Harvard are provided in Supporting Information*) serve as the template to secure approvals to share clinical data between health research institutions. Under these agreements, each institutional team loads medical facts into a locally controlled data repository that resides behind the hospital firewall.

#### Ethics Statement

The Institutional Review Boards (IRB) of the Beth Israel Deaconess Medical Center, Children's Hospital Boston, Dana-Farber Cancer Center, and Partners Health Care representing Massachusetts General Hospital and Brigham and Women's Hospital individually approved use of their data for the SHRINE network. The human studies committee (IRB) at Harvard Medical School in its role as fund administrator also reviewed and approved the SHRINE network. The regulatory committee of the Harvard CTSA (catalyst) developed a set of policies governing usage of the SHRINE network that was approved by the senior research vice president at each participating institution. Informed consent was not necessary as only aggregate numbers of patient attributes derived from medical records were provided, a usage considered non-human research by all IRBs.

### VI. Mapping Heterogeneous Medical Coding Systems in Multi-Site Studies

Ideally, every hospital would adopt the same standard set of medical concepts and relationships to record patient observations. However, different hospitals often have differing clinical information systems, medical coding practices, service specialties, and patient populations. Different investigator users and data managers often have differing perspectives on how clinical data should be schematically represented and semantically queried. Accounting for these differences can quickly exhaust the human resources available. SHRINE aims to maximize the breadth of supported research studies without requiring significant investment in human expert curators.

**Figure 4:**
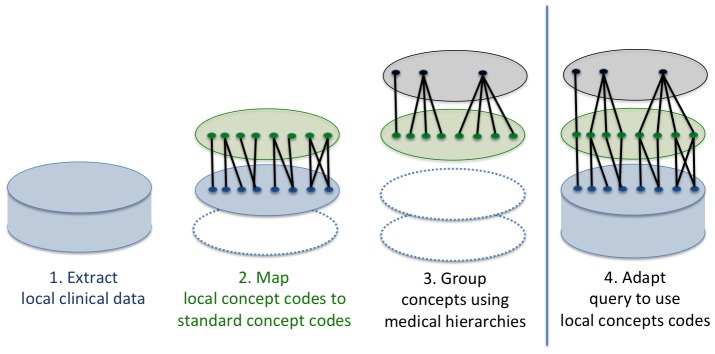
Hospital Data Mapping Scenario. *First*, existing clinical data are extracted into a locally controlled database for research. *Second*, each local code is mapped to one or more standard concept codes, and vice versa. *Third*, related medical concepts are grouped using standard hierarchies curated by medical experts. The bipartite graphs produced by this process enable bidirectional translation between concept systems. *Fourth*, adapt the incoming query to use the local concept codes.

**Figure 5:**
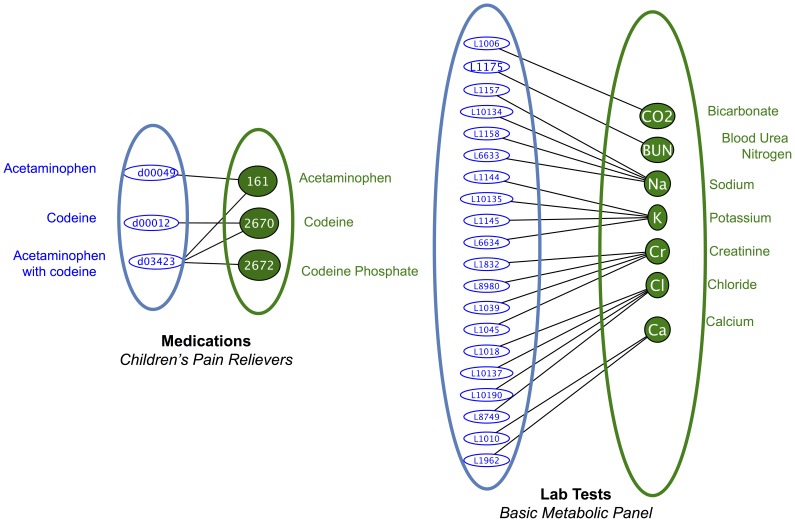
Constructing Bipartite graphs to map concept systems. ***Left*** *: Medications* are mapped between Children's Hospital Boston (blue) and the RxNorm standard (green) if they share a drug ingredient. The hospital concept code for Acetaminophen is mapped to the RxNorm concept code for Acetaminophen. Codeine also has one mapping. ‘Acetaminophen with Codeine’ has a mapping to RxNorm for each of its ingredients. Patients recorded with the local concept ‘Acetaminophen with Codeine’ will match standard queries using any of the mapped RxNorm drug ingredients. ***Right*** *: Lab Test concepts* are mapped between Children's Hospital Boston (blue) and the LOINC standard (green). Bicarbonate and Blood Urea Nitrogen are each mapped once. Other lab tests require a one-to-many mapping, for example, there are at least four different metabolic tests for sodium (Na+) levels recorded in the Children's Hospital Boston clinical systems.

**Figure 6:**
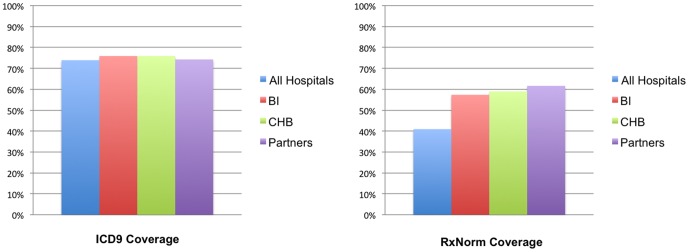
Percentage of Diagnosis and Medication concepts mapped for SHRINE queries at participating Harvard affiliated teaching hospitals. ***Left***: Percentage of ICD9-CM diagnoses concepts mapped to at least one diagnosis concept at the hospital. ***Right***: Percentage of RxNorm medication concepts mapped to at least one patient medication concept at the hospital.

Figure 4 illustrates the mapping scenario for a typical SHRINE participating site. *First*, the hospital extracts patient observations from various clinical databases into a locally controlled patient data repository. *Second*, hospital data curators construct bipartite graphs (key value pairs) for each of the four categories of clinical concepts defined in the Core Ontology. Each bipartite graph relates a set of local concepts to a set of standard concepts. Figure 5 contains mapping examples for lab tests and medications. Third, medically related concepts are grouped and their relationships are traversed using standard medical hierarchies. Fourth, the local hospital is now able to translate the incoming query to use local concept codes. Figure 6 reports the coverage of supported medication and diagnoses concepts at four Harvard hospitals. Figure S2 provides a screenshot of the software that enables the mapping process.

### VII. Adapting Network Queries for Local Execution

SHRINE *Adapters* are interfaces between the SHRINE network and the local patient data repository[43]. The Adapter translates incoming queries so that the query can be executed locally without changing the data in the local repository. Each participating SHRINE peer hosts an Adapter loaded with mappings that support query terms in the ‘Core Ontology’.

SHRINE Adapters validate, audit, translate, and anonymize queries. First, each Adapter validates that the query is from a trusted source by validating digital signature of the investigator identity[42]. Second, each Adapter audits the investigator to ensure against suspicious query activity such as excessive queries for the same small patient cohort. Third, the Adapter translates the query concepts into a format recognizable by the local data repository. Fourth, the Adapter anonymizes the patient count by applying a Guassian filter accurate to within +/−3 patients of the actual result[44]. Lastly, each Adapter responds to the originating SHRINE Broadcaster-Aggregator.

SHRINE provides a plug-in architecture allowing any data repository to be used so long as it accepts SHRINE messages. By default, SHRINE is configured to use the i2b2 data repository because it is commonly used[28], [45]. Institutions that use a third party data repository can participate in SHRINE by implementing the open messaging interface. Both the SHRINE[22] and i2b2[46] software packages are freely available and Open Source.

### VIII. Scaling to National Participation

**Figure 7:**
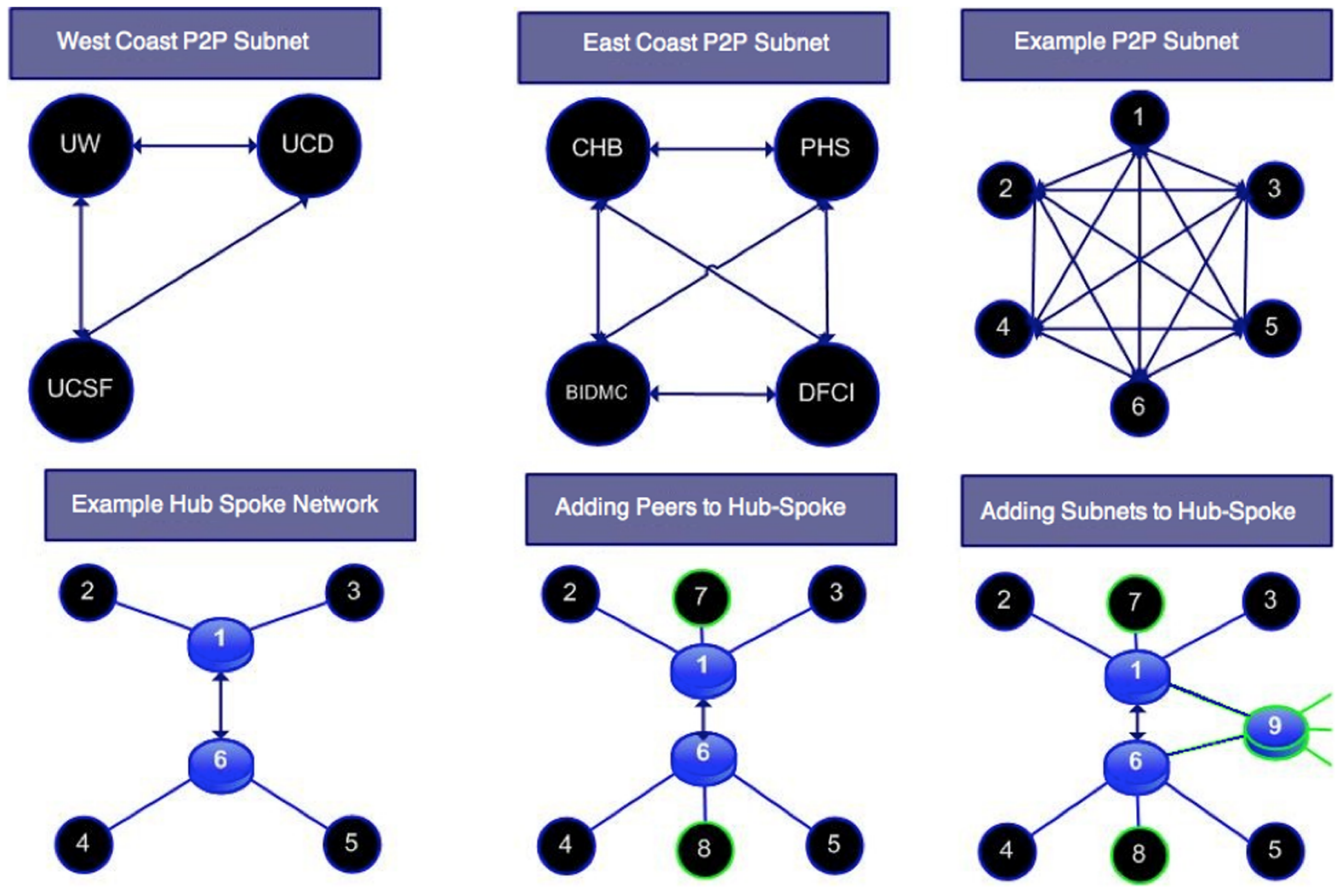
Peer Group configurations. *Top*: P2P networks are shown for the deployed West and East coast SHRINE networks with 3 and 4 peers respectively. P2P networks have n*(n-1)/2 edges. In the example p2p network with 6 peers, 6*5/2 = 15 edges are drawn. A 60 node P2P network would have 60*59/2 = 1,770 edges. *Bottom*: Hub Spoke networks are drawn starting with 6 peers. As peers are added, they can attach with a single link to an existing hub. As new hubs are formed regionally, they can be easily attached to the overall network.

**Figure 8:**
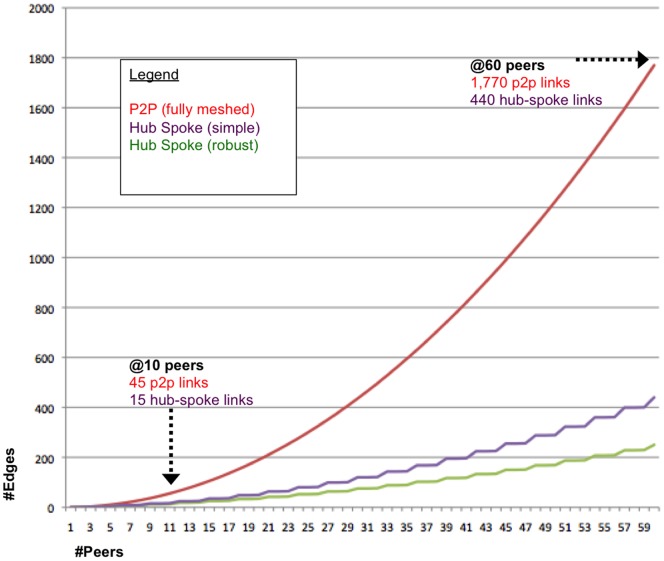
Quadratic growth in the number of edges in a communication network. Each edge incurs administrative overhead to maintain a list of peer locations and trust relationships. Fully meshed peer-to-peer (P2P) topologies have N*(N-1)/2 edges shown in red. Edge growth of hub-spoke topologies are shown with an average hub size of 3 (size of the first deployments of east and west coast networks). A simple hub-spoke topology requires one additional link per hub, shown in green. A fault tolerant topology requires two additional links per hub, shown in purple. With 60 peers, the number of p2p edges is administratively infeasible with 1,770 firewall rules and trust relationships.

Groups of SHRINE hospitals (peers) can be configured in peer-to-peer (p2p) or hub-spoke network topologies (Figure 7). In a p2p network, every peer has a link to every other peer. In relatively small SHRINE networks, p2p topologies can be configured with a few links. However, the number of direct links quickly grows with the number of peers in a fully-meshed network (Figure 8). Because each link requires a firewall rule and webservice URL, even a modestly sized network of 10 peers would require 45 firewall exceptions and 10 duplicate copies of routing information. In a network of 60 institutions, a p2p (fully meshed) network would require 1,770 firewall rules and 60 duplicate routing tables, which could overburden network administrators. Instead, larger deployments are more often arranged in hub-spoke topologies, as exemplified by the quickly deployed CARRAnet registry.

Similar to TCP/IP networks, SHRINE facilitates grouping regional peers into subnetworks and then links them together. This is highly desirable because communication networks typically grow according to power law[47], [48] naturally leading to community ‘hub’ formations. The hub-spoke deployment was previously utilized for the SPIN human tissue network, linking 7 large independent medical centers into regional peer groups [33].

SHRINE peers can participate in many different studies with many different institutions at the same time without changing the source data. Multiple Adapter mappings can be loaded for different study objectives. For example, the SHRINE *Core Ontology* (Table 2) was designed to provide maximal breadth of medical concepts commonly available in Electronic Health Record (EHR) systems. However, the core ontology does not describe data collected outside the EHR setting, such as patient registries or clinical trials. In such cases, it is necessary to adopt[49] or define ontologies suitable to how the data are collected. Because SHRINE translates the query rather than transforming the data, multiple study-specific views can occur simultaneously without source data duplication or transformation.

### **Discussion**

In this era of ‘translational’ research[50], [51], there is a growing and critical need for systems that streamline clinical data access for research while maintaining patient privacy and safety. Concurrently, the need for ever-larger cohort sizes[3], [31] increasingly necessitates crossing institutional boundaries between healthcare and research organizations that individually have insufficient numbers of patient-subjects. In reusing the by-products of routine care delivery, SHRINE has capitalized on low cost cohort identification with very large yields in terms of both number of patients and number of phenotypic features. SHRINE institutions and whole networks are increasingly being instantiated for population scale measurement on regional and national scales.

The widespread use and wide range of investigation scenarios served suggest that there may be broader applicability for other clinical research uses. Since SHRINE is more a network API rather than a final product, it is possible to envision new applications. For example, a European public-private partnership is evaluating the SHRINE platform to locate patient cohorts for clinical trials. There is also a strong potential for using SHRINE to locate human biospecimens for genomic studies[31], [52], [53], monitor population health [35], [54], and detect adverse medication events [36], [55], [56].

The authors recognize several limitations in this work. Limiting results to patient counts was essential in the building phases to reach agreement among hospital stakeholders. Consequently, extracting clinical details on selected patients is currently a manual process requiring IRB approval from each hospital and technical assistance. The next major development of SHRINE will focus on providing HIPAA Limited Data Sets on the subset of patients that match an IRB approved query such that the application process is streamlined for investigators.

Mapping medical concept dictionaries do not always produce perfect translations between concept systems. In the case of patient demographics and diagnoses, mappings were rather straight forward as billing standards were already in place. In the case of medications and lab results, mappings were much more difficult. Future work with the NCBO[49] aims to improve and increasingly automate our ability to map between coding systems.

Important study variables, such as smoking status[57], [58], co-morbidities[59], and family disease history[60] are often missing from the coded record and more likely to appear in physician notes. These variables can often be extracted[61], [62] using Natural Language Processing (NLP). A previous version of the SHRINE federated query protocol worked in this way[33] by searching pathology reports for human tissues[52] that matched coded clinical criteria[63]. However, at the time of this writing, NLP processing is not directly integrated into the SHRINE software. The adoption of enterprise-wide NLP processing tools such as cTAKES[62] may enable deeper and automatic extraction of data contained in unstructured text.

Biases in patient populations, medical coding practices, and records management directly influence which medical facts can be uniformly studied and how the results are interpreted. As the number of SHRINE participating peers and medical concepts increase, so too does the burden on an investigator. In response, we are exploring methods to empirically guide or ‘autosuggest’ features relevant to a particular disease study.

In conclusion, in an era where EHR implementation is growing rapidly, SHRINE provides a scalable solution for querying the informational byproducts of healthcare to conduct regional and national disease studies. SHRINE seeks to overcome problems of false discovery by 1) increasing the number of patients observed, 2) validating results across many patient settings, and 3) capturing the multitude of phenotypic characteristics observed during patient care. SHRINE is now operational at many participating institutions and is available open source. New institutions interested in sharing clinical data can use the SHRINE software and policy agreements, either in whole or in part (see Supporting Information). Because there is no central database, regional subnetworks and study specific collaborations are free to form independent of any organizing body. Current uses of SHRINE are primarily for locating patient cohorts and studying diseases at the population scale, with the possibility for many more investigation scenarios such as clinical trials preparation and genomic studies involving human specimens.

#### Related Work

Several other research efforts have sought to develop multi-site clinical research platforms. Each research network is designed for a specific investigator scenario, such as population health statistics[64], cancer informatics[65], biomedical imaging[66], and biomedical resource identification[66]. These efforts are also open source, with many years of shared history formalizing the policy agreements and developing the technical capabilities. Among these, SHRINE is most similar to other distributed population query efforts[67]. Twelve distributed population query systems (including SHRINE) are being independently evaluated to achieve the objectives defined by the Office of the National Coordinator, a complete comparison here is well beyond the scope of this report.

As a general clinical data integration platform, SHRINE is similar to other distributed query systems that use a mediated schema[68]. Mapping mediated schemas to heterogeneous local schemas is among the most challenging problems in computer science (AI-complete)[69]. SHRINE query translation is essentially synonym expansion, whereas other query mediators can fully rewrite the query to the source system[70]. Defining concept synonymy is often an easier problem to solve generally, suggesting that SHRINE may be easier to implement than other systems that provide more advanced query rewrite features.

## Supporting Information

Figure S1 **Federated Query Sequence.** **1**) Investigator starts query with the provided user credentials and query criteria. **2–3**) Investigator credentials are certified and digitally signed. **4**) Query is broadcast to all trusted peers. **5–6**) Each Adapter validates the digitally signed identity and translates the criteria. **7**) Each Adapter queries their local Patient Data Repository. Most investigators will only receive the patient set size (count). Some investigators (national disease registry) can see additional data. **8–9**) Results are asynchronously aggregated. **10**) Aggregated results shown to investigator.
(TIFF)

Figure S2 **Screenshot of Mapping Tool (SHRIMP).** *Left*: Children's Hospital Boston Medication fragment is selected and focused on propranolol (a beta blocker). *Top Middle*: concept details including local key and name are displayed, which defines how this medication is coded at CHB. *Top Right*: the local concept code for propranolol is mapped to two core concepts: propranolol (the brand name drug) and propranolol hydrochloride (the generic drug). The hospital concept and the core concept refer to have the same ingredient. *Bottom*: Users can quickly search the core concepts to find mappings for the hospital concepts.
(TIFF)

Information S1 **SHRINE Business Rules.** This supporting information includes a set of operating principles or ‘Business Rules’ that were agreed upon by all institutions participating in the Harvard network. The business rules can be used in whole or in part to build agreement for new SHRINE networks.
(DOC)

Information S2 **Technical Supporting Information.** The Technical Supporting Information describes requirements and experiences using different data repositories and mapping different medical coding systems. This SI also includes a list of SHRINE query capabilities supported in the Core Ontology.
(DOCX)
