# Supplementary figures and images for: SHRINE: Enabling Nationally Scalable Multi-Site Disease Studies
Source: PLoS One. 2013 Mar 7;8(3):e55811. doi: 10.1371/journal.pone.0055811 (PMC3591385; doi:10.1371/journal.pone.0055811)

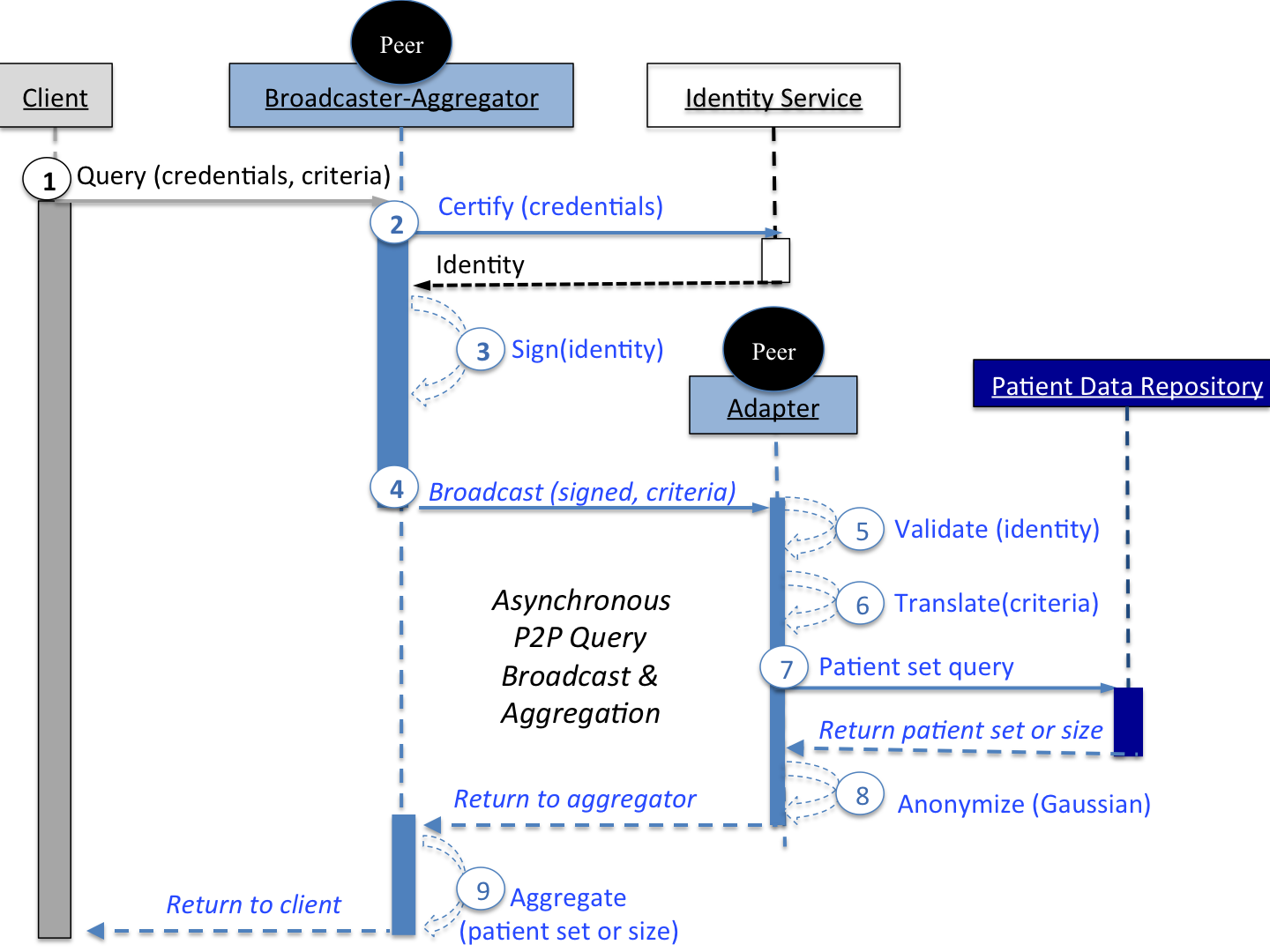

Supplement: Figure S1 — Federated Query Sequence. 1) Investigator starts query with the provided user credentials and query criteria. 2–3) Investigator credentials are certified and digitally signed. 4) Query is broadcast to all trusted peers. 5–6) Each Adapter validates the digitally signed identity and translates the criteria. 7) Each Adapter queries their local Patient Data Repository. Most investigators will only receive the patient set size (count). Some investigators (national disease registry) can see additional data. 8–9) Results are asynchronously aggregated. 10) Aggregated results shown to investigator. (TIFF) [file pone.0055811.s001.tiff]

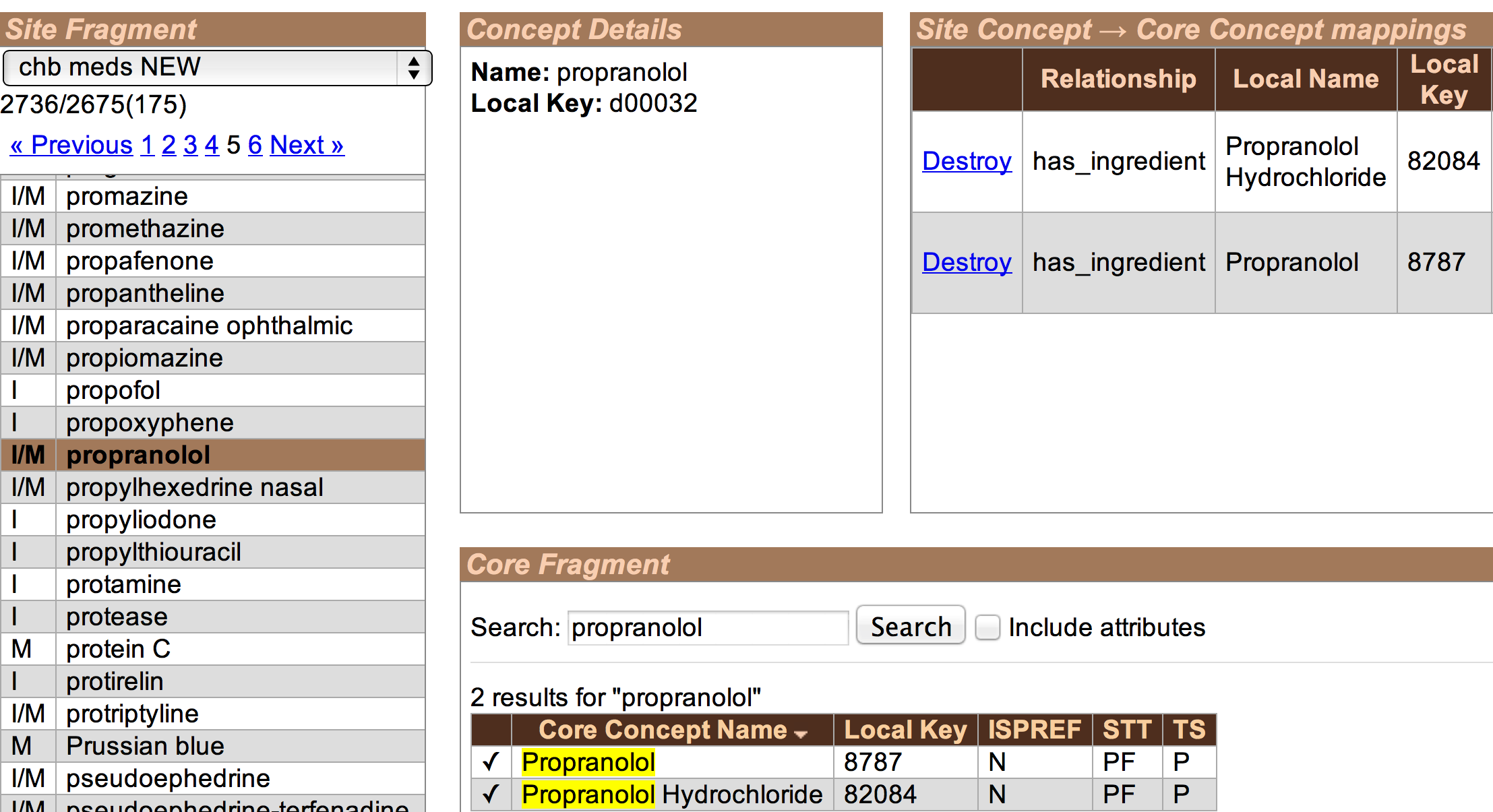

Supplement: Figure S2 — Screenshot of Mapping Tool (SHRIMP). Left: Children's Hospital Boston Medication fragment is selected and focused on propranolol (a beta blocker). Top Middle: concept details including local key and name are displayed, which defines how this medication is coded at CHB. Top Right: the local concept code for propranolol is mapped to two core concepts: propranolol (the brand name drug) and propranolol hydrochloride (the generic drug). The hospital concept and the core concept refer to have the same ingredient. Bottom: Users can quickly search the core concepts to find mappings for the hospital concepts. (TIFF) [file pone.0055811.s002.tiff]
