# Supplementary material for: SHRINE: Enabling Nationally Scalable Multi-Site Disease Studies
Source: PLoS One. 2013 Mar 7;8(3):e55811. doi: 10.1371/journal.pone.0055811 (PMC3591385; doi:10.1371/journal.pone.0055811)
Supplement: Information S1 — SHRINE Business Rules. This supporting information includes a set of operating principles or ‘Business Rules’ that were agreed upon by all institutions participating in the Harvard network. The business rules can be used in whole or in part to build agreement for new SHRINE networks. (DOC) [file pone.0055811.s003.doc]

SHARING CLINICAL DATA ACROSS HARVARD CATALYST INSTITUTIONS FOR RESEARCH PURPOSES

Business Policies

This document provides a framework intended to insure that the primary goal of SHRINE – catalyzing research collaborations among Harvard investigators by sharing clinical data across the participating institutions - is realized in an ethical, respectful and transformative fashion. Adoption of these principles will drive technical solutions to insure that each is properly implemented in the most efficacious and reliable manner. The first phase of SHRINE will provide access only to aggregate summary data counts (“start-up”); subsequent phases will follow as technology development and institutional approvals allow. These Business Rules have been approved by the Senior Vice Presidents for Research at the Participating SHRINE Organizations.

1. Access to Data

- Access to data is limited to “Qualified Faculty” only, herein defined as Harvard Medical School faculty appointees who are both
  - Appointed at or above Instructor level and
  - *Employees* of and primarily appointed by those institutions who have agreed to share their data, e.g. *only* Qualified Faculty from institutions who have agreed to share their data may participate in the SHRINE network
- Harvard Faculty from non-health care Harvard Catalyst institutions (such as HMS, HSPH, SEAS, HBS) who do not produce data but who wish to access data for research purposes must collaborate with an appropriate (discipline specific) Qualified Faculty member from one or more of the participating SHRINE hospitals.
- Access to Aggregate Data (summary numbers of patients in category of interest) by Qualified Faculty will not require IRB review.
- Access by academics from institutions outside of the Harvard Catalyst institutions will not be allowed in the start up phase of SHRINE.
- Collaborations between Qualified Faculty and industry requiring access to federated data will not be allowed in the start up phase.
- Termination of the start up phase and/or initiation of a subsequent phase will require explicit approval of each participating institution.

2. Specific Limitations on Data Access and Use

- Access to each approved query topic will be limited to a six month period following first release of data.
- Sample sizes smaller than 10 will not be returned in order to prevent inadvertent identification of the sampled patients.
- All appropriate state and federal laws and regulations governing specially protected information will apply.

3. Insuring Integrity of Data Use

- All Qualified Faculty, and their collaborating investigators must complete and sign the SHRINE Terms of Access Agreement before making a data request; this module will contain the following elements:
  - - Acknowledgement of principles regarding ethical considerations in using shared clinical data;
    - Statement prohibiting any attempt to identify any individual patient;
    - Appropriate language regarding protection of intellectual property;
    - Publication policy (regarding use of hospital name only; see below)
    - Statement of penalty for violating agreement.
- All data requests will be reviewed by the designated representative(s) of the Harvard Catalyst leadership (“Harvard Catalyst Data Steward) for scientific and operational integrity. The Data Steward will have the authority to approve, disapprove or defer requests; this review should take no longer than two business days unless an extended dialogue is warranted.
- All data queries will be archived and can be included, by request, in the quarterly reporting to the institutional Senior Vice Presidents of Research.
- The Harvard Catalyst Data Steward will periodically audit individual data queries for compliance with the originally approved request.
- Publications in which data sources (hospital) are identified by name will be reviewed for use of name only by each identified hospital prior to submission. Requests for use of name will be sent to the institution Senior Vice President of Research or their designee. Any hospital which does not agree to be identified by name as a data source will be instead identified as a “Harvard-affiliated hospital”.
- A Harvard Catalyst Data Use Committee will be constituted by the Catalyst Regulatory Committee and will resolve requests not covered by existing policies; membership will include all participating hospitals, Harvard Catalyst leadership, the Data Steward and the Harvard Catalyst Regulatory Officer.
- A report of all approved SHRINE queries and associated system use will be provided by the Harvard Catalyst Data Steward to participating SHRINE institutions on a quarterly basis.
- A process for managing inquiries and/or concerns regarding access to patient data will be developed in consultation with the Senior Vice Presidents.
