# Supplementary material for: SHRINE: Enabling Nationally Scalable Multi-Site Disease Studies
Source: PLoS One. 2013 Mar 7;8(3):e55811. doi: 10.1371/journal.pone.0055811 (PMC3591385; doi:10.1371/journal.pone.0055811)
Supplement: Information S2 — Technical Supporting Information. The Technical Supporting Information describes requirements and experiences using different data repositories and mapping different medical coding systems. This SI also includes a list of SHRINE query capabilities supported in the Core Ontology. (DOCX) [file pone.0055811.s004.docx]

TECHNICAL SUPPORTING INFORMATION

**Patient Data Repository**

- SHRINE is not bound to any specific patient data repository schema or software.
- SHRINE is most often installed with an i2b2 Patient Data Repository[[1](#_ENREF_1), [2](#_ENREF_2)].
- SHRINE has been used with custom patient data repositories in production settings.

Minimum data elements

A custom patient data repository that responds to SHRINE patient queries must have 3 data elements per fact: a patient number, concept code, and observation timestamp. Adding an encounter number is necessary to support queries based on encounter. At this time, encounter queries are not used in SHRINE deployments, in part because hospitals may not agree on what constitutes an encounter (patient visit). *Note*: developing a patient data repository can be very challenging, and will probably require more effort than adopting an existing implementation.

Effort

Using the I2b2 data repository has lead to faster deploy times. The time required to deploy and populate the repository varies[[3](#_ENREF_3)]. Most of the effort is in data preparation. Implementing the messaging interface is more challenging, but it has been done several times before in different languages such as C#, Java, etc. The XML query adapter interfaces can be implemented in any language. Opening a firewall port for the system requires obtaining permission from the local hospital network admins.

**Mapping Tool (SHRIMP)**SHRIMP is a simple mapping program for creating associations between two sets of provided data. SHRIMP was used to create associations between the *Core Ontology* and datasets at various hospitals. SHRIMP facilitated the mapping of nearly all concepts in the Core Ontology to concepts at 5 large hospitals in Boston. Most importantly, this was achieved with only one dedicated clinical data curator, suggesting that SHRIMP is not resource intensive.

Inputs to SHRIMP are generally *fragments* and *mappings*. *Fragments* define the set of concept codes supported by an institution for a given concept category. For example: “Children’s Hospital Boston Medications” is one fragment. *Mappings* define the relationships between the local hospital fragment and the standard core fragment. See figure SF2.

SHRIMP outputs individual mappings files and a merged Adapter Mapping XML file that contains all hospital supported concept codes and hierarchical relationships. At runtime, adapter mappings easily fit in memory and provide constant time concept translation functions.

**Mapping Effort and Experiences in the Harvard SHRINE Network**

Diagnoses codes were already standardized for administrative billing so the standard was adopted without modification. Demographic codes were not standardized but were nevertheless trivial to associate using synonyms. Lab tests and medication codes are much more difficult to map and require manual inspection by a domain expert, see report by Matvey Palchuck et al[[4](#_ENREF_4)]. Some lab tests have a one-to-one mapping, for example the Blood Urea Nitrogen lab test at Children’s Hospital Boston. Other lab tests require a one-to-many mapping, for example, there are at least four different metabolic tests for sodium (Na+) levels recorded in the Children’s Hospital Boston clinical systems. Medications are even more difficult to map, especially brand name combination drugs such as Tylenol with Codeine. SHRINE medications are mapped once for each generic ingredient. This means that Tylenol with Codeine is mapped once to Acetaminophen and once to Codeine Phosphate. From this perspective, patients prescribed Tylenol with Codeine are equivalent to patients who were separately prescribed Acetaminophen and Codeine Phosphate.
 **Technical Requirements**SHRINE runtime requires a java web server to host the application (the default is Tomcat 6 and JDK6). X509 Certificates and JKS keystores provide PKI trust for secure HTTPS communication. SHRINE signatures conform to the W3C standard for XML digital signatures. SHRINE messages are web services that can be implemented in any language. At runtime, each SHRINE installation is configured as a broadcaster-aggregator, query adapter, or both.

**Supported Query Features Using the Core Ontology**

Query features are listed below in a format that is loosely an extended BNF grammar. This is catalog of SHRINE patient query features are specific to Core Ontology, and will be extended in future releases. Concepts from each coding system in the Core Ontology are “quoted”, as it would otherwise be impractical to list many thousands of concepts. The symbol “+” means at least once, “*” means 0 or more times, and “|” denotes an OR condition. The query definition contains at least one query panel. Each panel is displayed as a query group in the user interface. The result of the SHRINE query is the set of patients that match the criteria defined in all of the query panels. The query result is either the size of the patient set (count) or the individual facts in the patient set. Each query panel optionally contains constraints on or more query concepts. The panel constraint can be the number of times the concept was uniquely recorded, for example, at least two diagnoses of essential hypertension. The panel constraint can refer to an interval of time, bounded by a minimum and maximum date the observation fact was recorded. The panel constraint could be inverted, meaning find all patients who do not have the concepts in the panel. Min and max are denoted by the “>” and “<” signs for clarity. The concepts used in a query are from the ontology, in this case the SHRINE Core Ontology (Other ontologies can and have been used). A concept refers to a demographic, diagnosis, medication, or lab test. A demographic concept can either be a gender, a race/ethnicity code, a language, a marital status, or a patient age. Each of these demographic terms are defined using the standards defined by the ontology. Diagnoses and medication concepts can be grouped to enable hierarchical querying. Lab tests can also be queried by value, to see if the lab test was low, average, or high.

Query Definition

query_definition = query_panel+

query_panel = panel_constraint* concept+

panel_constraint =  occurrences | time_interval | invert

occurrence = (min | max) number

time_interval = (min | max) date

invert = boolean

min = “>”

max = “<”

concept = demographic | diagnosis | medication | labtest

demographic = gender | race_ethnicity | language | marital_status | patient_age

gender = “HL7 Administrative Gender”

race_ethnicity = “CDC race & ethnicity code sets”

language = “ISO 639-1 language codes”

marital_status = “HL7 marital status”

patient_age = patient_age_range | patient_age_value

patient_age_range = patient_age_value+

patient_age_value = number

diagnosis = diagnosis_group | diagnosis_code

diagnosis_group = “CCS multi-level diagnosis grouping”

diagnosis_code = “ICD9-CM diagnoses code”

medication = medication_group | medication_code

medication_group = “NDF-RT multi-level drug grouping”

medication_code = “RxNorm drug ingredient code”

labtest = labtest_constraint? labtest_code

labtest_constraint = constraint_value | constraint_flag

constraint_value = (min | max) number

constraint_flag = low | normal | high

labtest_code = “LOINC code”

**REFERENCES**

1. Nalichowski, R., et al., *Calculating the benefits of a Research Patient Data Repository.* AMIA ... Annual Symposium proceedings / AMIA Symposium. AMIA Symposium, 2006: p. 1044.

2. *i2b2 Open Source Software Website. Available at https://*<http://www.i2b2.org/software/>*. Accessed 2013, Jan 5.* .

3. Majeed, R.W. and R. Rohrig, *Automated Realtime data Import for the i2b2 Clinical data Warehouse: Introducing the HL7 ETL cell.* Studies in health technology and informatics, 2012. **180**: p. 270-4.

4. Palchuk, M.B., et al., *Enabling Hierarchical View of RxNorm with NDF-RT Drug Classes.* AMIA ... Annual Symposium proceedings / AMIA Symposium. AMIA Symposium, 2010. **2010**: p. 577-81.
